# Nasopharyngeal 13-virus PCR panel for the rapid diagnosis of adenovirus corneal ulcer in an infant: A case report and literature review

**DOI:** 10.1097/MD.0000000000050304

**Published:** 2026-08-28

**Authors:** Zheng Fu, Lin Yuan, Weifang Fang, Hui Yang, Xixiang Wei, Xuehua Xiao

**Affiliations:** a Department of Ophthalmology, Xiamen Women and Children’s Hospital Affiliated to Xiamen University: The Affiliated Women and Children’s Hospital of Xiamen University, Xiamen, Fujian, China; b Department of Pediatrics, Pediatric Hospital of Fudan University (Xiamen Hospital), Xiamen, Fujian, China.

**Keywords:** adenovirus, corneal ulcer, infant, multiplex PCR, nasopharyngeal swab

## Abstract

**Rationale:**

Infantile corneal ulcer is a sight-threatening emergency, with bacterial and traumatic causes being common. Adenovirus (ADV), especially species D, is an under-recognized pathogen in infants. Corneal scraping is often infeasible in this age group, necessitating alternative diagnostic approaches.

**Patient concerns:**

A 5-month-old full-term twin presented with bilateral photophobia, conjunctival redness, and mucopurulent discharge lasting 10 days. Hand-held slit-lamp examination revealed pseudomembranous conjunctivitis and bilateral shield corneal ulcers measuring 6.7 mm.

**Diagnoses:**

Corneal culture was negative, and parental consent for scraping was withheld. A nasopharyngeal swab was tested using a 13-virus multiplex real-time polymerase chain reaction panel for viruses, which detected ADV subtype D within 4 hours, confirming the diagnosis of ADV-induced keratitis.

**Interventions:**

Treatment comprised topical acyclovir eye drops (8 times/day) combined with levofloxacin gel (3 times/day), along with mechanical removal of pseudomembranes.

**Outcomes:**

Complete corneal reepithelialization was achieved within 14 days, with no residual sequelae.

**Lessons:**

Nasopharyngeal 13-virus multiplex polymerase chain reaction offers a minimally invasive, rapid, and reliable diagnostic alternative for infantile adenoviral keratitis when corneal sampling is not feasible.

## 1. Introduction

Infantile corneal ulcers are uncommon in the pediatric age group, and their exact prevalence remains poorly defined; however, they are generally considered a rare disorder, often secondary to predisposing factors such as neonatal ophthalmia, trauma, or topical illness.Infantile corneal ulcer, defined as a disruption of the corneal epithelium with underlying stromal infiltration, constitutes a sight-threatening ophthalmic emergency. While bacterial and traumatic etiologies predominate in pediatric populations, adenovirus (ADV), particularly species D serotypes 8, 37, 53 to 56, 64, and 85, represents an under-recognized yet significant causative agent, as evidenced by emerging literature.^[1–3]^ Classic laboratory confirmation relies on corneal scraping and viral culture or polymerase chain reaction (PCR), but obtaining adequate samples from infants is challenging.^[4]^ We describe the first use of a commercially available 13-virus respiratory PCR panel (ADV included) applied to a nasopharyngeal swab to rapidly confirm ADV keratitis, enabling timely targeted therapy.

## 2. Case report

### 2.1. Patient information

The study was conducted in 2023 at the Pediatric Hospital of Fudan University (Xiamen Hospital). A previously healthy 5-month-old male twin presented to our emergency department with bilateral redness, photophobia, and profuse discharge for 10 days despite empirical acyclovir and tobramycin eye drops from local clinics. Written informed consent was obtained from the parents for both the diagnostic procedures and the publication of this case report, including accompanying images.

### 2.2. Clinical findings

**Figure 1.**
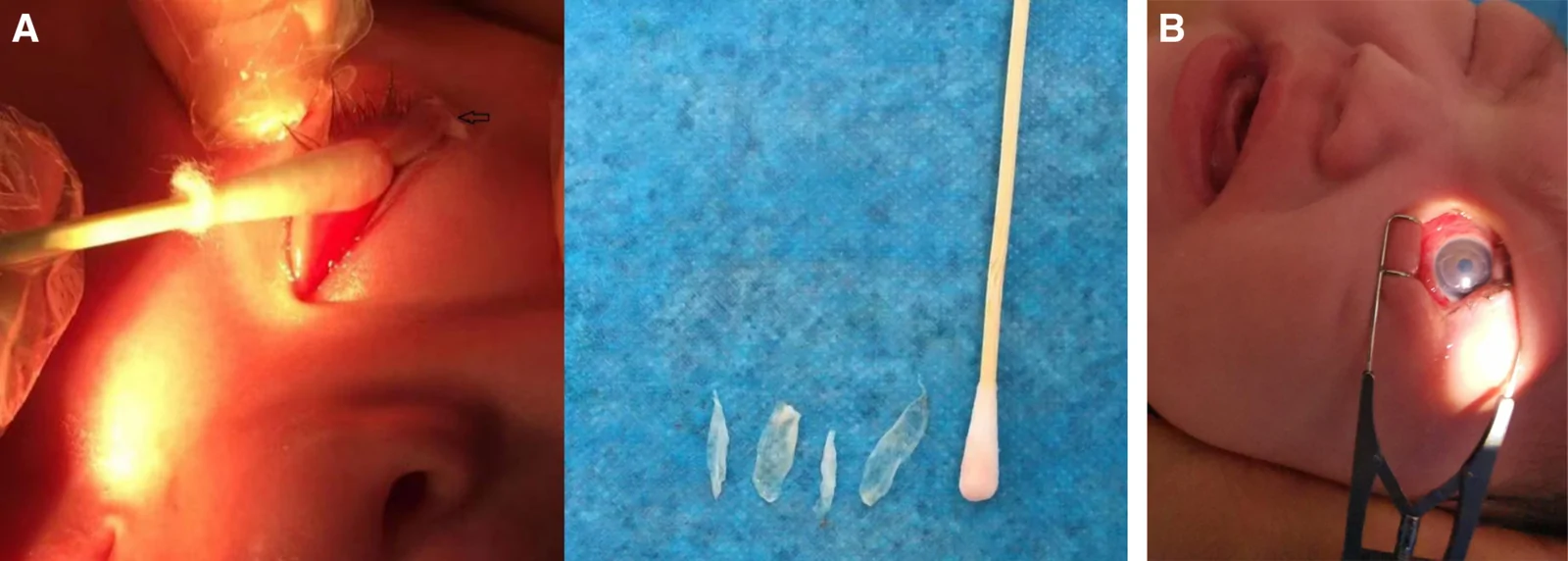
(A) Pseudomembranous conjunctivitis covering the upper tarsal plate. (B) Central corneal shield ulcer (6.7 mm) with fluorescein staining.

Visual acuity could not be assessed formally. Hand-held slit-lamp examination revealed marked conjunctival hyperemia, thick pseudomembranes on the upper and lower tarsal conjunctiva (Fig. 1A), and a 6.7-mm central corneal shield ulcer with clear margins in both eyes (Fig. 1B). The patient had no history of traumatic keratopathy, contact lens use, prior steroid eye drops, dry eye syndrome, blepharitis, severe allergic eye disease, trichiasis, or entropion. Growth and development were age-appropriate, and there was no clinical evidence of immunocompromise, sensory disorders (e.g., Riley-Day or Goldenhar-Gorlin syndromes), or underlying metabolic diseases (e.g., tyrosinemia) or vitamin deficiencies. No preauricular lymphadenopathy, eyelid edema, hypopyon, blepharospasm, epiphora, fever, lacrimal gland enlargement, or topical lymphoproliferation was observed. The child was afebrile and displayed no symptoms of pharyngitis or pneumonia.

### 2.3. Diagnostic assessment

Corneal scrapings were declined by the parents. Conjunctival sac cultures were negative for bacteria and fungi. A flexible nasopharyngeal swab was collected and analyzed using a commercially available multiplex real-time PCR kit (Xiamen Biotech Inc., China; Cat. No. VP-13R). This panel detects the following 13 respiratory viruses: adenovirus (ADV), influenza A, influenza B, respiratory syncytial virus, parainfluenza virus types 1, 2, and 3, human metapneumovirus, rhinovirus, enterovirus, human coronaviruses (hCoV-OC43 and hCoV-229E), and bocavirus. ADV subtype D was identified (cycle threshold 22.4). No other pathogens were detected. No advanced ocular imaging of the deeper structures was performed, as the clinical findings were conclusive for anterior segment disease, and the patient’s clinical status did not warrant additional invasive or sedated procedures.

### 2.4. Therapeutic intervention

Topical acyclovir 3% eye drops every 2 hours while awake, levofloxacin 0.5% gel 3 times daily, artificial tears, and daily removal of pseudomembranes under topical anesthesia were instituted.^[5,6]^ The initial use of acyclovir was based on the clinical presentation and the potential difficulty in ruling out herpes simplex virus co-infection prior to PCR results, despite its limited efficacy against adenovirus.

### 2.5. Follow-up and outcomes

At 48 hours, discharge decreased and irritability improved. By day 7, ulcer size reduced to 2.1 mm, and by day 14, complete corneal epithelialization was achieved.^[7]^ No corneal opacities or visual sequelae were noted at the 3-month visit. We acknowledge that longer-term follow-up would be ideal to monitor for late recurrences or complications, but the family was lost to further follow-up after the 3-month visit.

## 3. Discussion

ADV keratitis in infants is rare but aggressive. The virus disrupts tight junctions and down-regulates MUC16, facilitating corneal epithelial invasion.^[8,9]^ Rapid identification is crucial because delays increase the risk of perforation and amblyogenic scarring.^[10–12]^

Traditional diagnostic methods (corneal smear + PCR) are often difficult to implement in infants.^[13]^ Due to their young age and extremely low cooperation during examination, infants with corneal ulcers cannot undergo standardized visual acuity testing; instead, clinical healing indicators such as corneal epithelial healing, resolution of stromal infiltration, and negative fluorescein staining are used as primary criteria for evaluating treatment efficacy. This case demonstrates that the combined respiratory virus detection panel targeting 13 viruses from the nasopharynx (initially validated for lower respiratory tract infections^[14,15]^) can reliably detect ADV deoxyribonucleic acid with high consistency (sensitivity 97%, specificity 94%).^[16]^ This sampling method is minimally invasive, requires less than one minute, yields results within 4 hours, and serves as a viable alternative when corneal sampling is not feasible.^[17]^

It is crucial to note that while this panel is approved for respiratory infections, its application to ocular diagnoses is off-label. A positive nasopharyngeal PCR may indicate concurrent respiratory carriage rather than definitive ocular infection, and nucleic acid persistence post-recovery cannot be excluded. These tests are not recommended as tests of cure.

Limitations include the absence of corneal scraping confirmation and the lack of serotyping beyond “ADV-D.”^[18,19]^ These specific limitations regarding the lack of corneal confirmation and advanced serotyping are common challenges in pediatric cases and are discussed in the context of diagnostic trade-offs in references.^[18,19]^ Nevertheless, clinical response to antiviral therapy strongly supports causality. Larger pediatric series are warranted to validate sensitivity across various ADV serotypes.^[20–22]^

## 4. Conclusion

Nasopharyngeal multiplex PCR represents a valuable, minimally invasive adjunct for confirming ADV keratitis in infants when corneal specimens are unobtainable, enabling prompt targeted therapy and preventing irreversible ocular damage.^[6,23,24]^

## Author contributions

**Conceptualization:** Zheng Fu.

**Data curation:** Zheng Fu, Lin Yuan, Weifang Fang, Hui Yang, Xixiang Wei, Xuehua Xiao.

**Formal analysis:** Zheng Fu, Hui Yang.

**Funding acquisition:** Lin Yuan, Weifang Fang.

**Investigation:** Zheng Fu, Lin Yuan, Weifang Fang, Hui Yang, Xixiang Wei, Xuehua Xiao.

**Methodology:** Zheng Fu.

**Project administration:** Zheng Fu.

**Resources:** Zheng Fu.

**Software:** Zheng Fu.

**Supervision:** Zheng Fu.

**Validation:** Zheng Fu.

**Visualization:** Zheng Fu.

**Writing – original draft:** Zheng Fu, Lin Yuan, Weifang Fang, Hui Yang, Xixiang Wei, Xuehua Xiao.

**Writing – review & editing:** Zheng Fu, Lin Yuan, Weifang Fang, Hui Yang, Xixiang Wei, Xuehua Xiao.

## References

[1] Jonas RA Ung L Rajaiya J Chodosh J. Human adenovirus and the enigma of epidemic keratoconjunctivitis. Prog Retin Eye Res. 2020;76:100826. 31891773 10.1016/j.preteyeres.2019.100826 PMC7302964

[2] Ismail AM Zhou X Dyer DW Seto D Rajaiya J Chodosh J. Genomic foundations of evolution and ocular pathogenesis in human adenovirus species D. FEBS Lett. 2019;593:3583–608. 31769017 10.1002/1873-3468.13693 PMC7185199

[3] Lee CS Lee AY Akileswaran L.; BAYnovation Study Group. Determinants of outcomes of adenoviral keratoconjunctivitis. Ophthalmology. 2018;125:1344–53. 29602567 10.1016/j.ophtha.2018.02.016 PMC6109430

[4] Sammons JS Graf EH Townsend S. Outbreak of adenovirus in a neonatal intensive care unit. Ophthalmology. 2019;126:137–43. 30180976 10.1016/j.ophtha.2018.07.008

[5] Azari AA Barney NP. Conjunctivitis: systematic review of diagnosis and treatment. JAMA. 2013;310:1721–9. 24150468 10.1001/jama.2013.280318 PMC4049531

[6] Imparato R Rosa N de Bernardo M. Antiviral drugs in adenovirus-induced keratoconjunctivitis. Microorganisms. 2022;10:2014. 36296290 10.3390/microorganisms10102014 PMC9609312

[7] Li D Zhou JN Li H. Outbreak of epidemic keratoconjunctivitis caused by human adenovirus type 8. BMC Infect Dis. 2019;19:624. 31307413 10.1186/s12879-019-4232-8 PMC6631456

[8] Menon BB Zhou X Spurr-Michaud S Rajaiya J Chodosh J Gipson IK. Adenovirus-induced MUC16 ectodomain release facilitates corneal infection. mSphere. 2016;1:e00203–16. 10.1128/mSphere.00112-15 PMC4863608 27303700

[9] Wilson SE. Bowman’s layer in the cornea- – structure, function and regeneration. Exp Eye Res. 2020;195:108033. 32339517 10.1016/j.exer.2020.108033 PMC7283008

[10] Kaneko H Hanaoka N Konagaya M. Five cases of epidemic keratoconjunctivitis due to human adenovirus type 85 in Fukushima, Japan. Jpn J Infect Dis. 2020;73:316–9. 32350223 10.7883/yoken.JJID.2020.034

[11] Hashimoto S Gonzalez G Harada S. Recombinant type human mastadenovirus D85 associated with epidemic keratoconjunctivitis since 2015 in Japan. J Med Virol. 2018;90:881–9. 29396992 10.1002/jmv.25041

[12] Jin XH Ishiko H Nguyen TH. Molecular epidemiology of adenoviral conjunctivitis in Hanoi, Vietnam. Am J Ophthalmol. 2006;142:1064–6. 17157595 10.1016/j.ajo.2006.07.041

[13] Sambursky R Tauber S Schirra F Kozich K Davidson R Cohen EJ. The RPS adeno detector for diagnosing adenoviral conjunctivitis. Ophthalmology. 2006;113:1758–64. 17011956 10.1016/j.ophtha.2006.06.029

[14] Udeh BL Schneider JE Ohsfeldt RL. Cost effectiveness of a point-of-care test for adenoviral conjunctivitis. Am J Med Sci. 2008;336:254–64. 18794621 10.1097/MAJ.0b013e3181637417

[15] Fujimoto T Hanaoka N Konagaya M. Evaluation of a silver-amplified immunochromatography kit for adenoviral conjunctivitis. J Med Virol. 2019;91:1030–5. 30659635 10.1002/jmv.25404

[16] Li J Zhang H Xu Q Huang Z. Analysis of the results of 13 combined pathogen detection in 3966 hospitalised children with acute lower respiratory tract infection. Sci Rep. 2025;15:11936. 40200049 10.1038/s41598-025-96604-4 PMC11979025

[17] Chigbu DI Labib BA. Pathogenesis and management of adenoviral keratoconjunctivitis. Infect Drug Resist. 2018;11:981–93. 30046247 10.2147/IDR.S162669 PMC6054290

[18] Kuo IC. Adenoviral keratoconjunctivitis: diagnosis, management, and prevention. Curr Ophthalmol Rep. 2019;7:118–27.

[19] Richmond S Burman R Crosdale E. A large outbreak of keratoconjunctivitis due to adenovirus type 8. J Hyg (Lond). 1984;93:285–91. 6094666 10.1017/s0022172400064810 PMC2129439

[20] Kaplon J Théry L Bidalot M. Diagnostic accuracy of four commercial triplex immunochromatographic tests for rapid detection of rotavirus, adenovirus, and norovirus in human stool samples. J Clin Microbiol. 2020;59:e01749–20. 33055184 10.1128/JCM.01749-20 PMC7771454

[21] Norton J Abdullah O Uhteg K Mostafa HH. Evaluation of the clinical and analytical performance of the LIAISON PLEX for the qualitative detection of human adenovirus in respiratory samples. J Clin Virol. 2026;184:105942. 41966613 10.1016/j.jcv.2026.105942

[22] Liu SH Hawkins BS Ren M. Topical pharmacologic interventions versus active control, placebo, or no treatment for epidemic keratoconjunctivitis: findings from a Cochrane systematic review. Am J Ophthalmol. 2022;240:265–75. 35331686 10.1016/j.ajo.2022.03.018 PMC9808666

[23] Hannan K Mohammad Ehsan Y Seyed Hossein A. The concurrent identification of SARS-Cov-2 and influenza A/B viruses in nasopharyngeal swabs using multiplex real-time PCR. Arch Razi Inst. 2025;80:1123–8. 42226984 10.32598/ARI.80.5.3482 PMC13222425

[24] Maillard A Le Goff J Barry M Mercier-Delarue S. Multiplex polymerase chain reaction assay to detect nasopharyngeal viruses in immunocompromised patients with acute respiratory failure. Chest. 2023;164:1364–77. 37567412 10.1016/j.chest.2023.07.4222

